# Mechanically Stretched Mesenchymal Stem Cells Can Reduce the Effects of LPS-Induced Injury on the Pulmonary Microvascular Endothelium Barrier

**DOI:** 10.1155/2020/8861407

**Published:** 2020-10-30

**Authors:** Jin-ze Li, Shan-shan Meng, Xiu-Ping Xu, Yong-bo Huang, Pu Mao, Yi-min Li, Yi Yang, Hai-bo Qiu, Chun Pan

**Affiliations:** ^1^Jiangsu Provincial Key Laboratory of Critical Care Medicine, Department of Critical Care Medicine, Zhongda Hospital, School of Medicine, Southeast University, Nanjing 210009, China; ^2^The State Key Laboratory of Respiratory Disease, Guangzhou Institute of Respiratory Disease, Department of Critical Care Medicine, The First Affiliated Hospital of Guangzhou Medical University, Guangzhou 510120, China

## Abstract

Mesenchymal stem cells (MSCs) may improve the treatment of acute respiratory distress syndrome (ARDS). However, few studies have investigated the effects of mechanically stretched -MSCs (MS-MSCs) in *in vitro* models of ARDS. The aim of this study was to evaluate the potential therapeutic effects of MS-MSCs on pulmonary microvascular endothelium barrier injuries induced by LPS. We introduced a cocultured model of pulmonary microvascular endothelial cell (EC) and MSC medium obtained from MSCs with or without mechanical stretch. We found that Wright-Giemsa staining revealed that MSC morphology changed significantly and cell plasma shrank separately after mechanical stretch. Cell proliferation of the MS-MSC groups was much lower than the untreated MSC group; expression of cell surface markers did not change significantly. Compared to the medium from untreated MSCs, inflammatory factors elevated statistically in the medium from MS-MSCs. Moreover, the paracellular permeability of endothelial cells treated with LPS was restored with a medium from MS-MSCs, while LPS-induced EC apoptosis decreased. In addition, protective effects on the remodeling of intercellular junctions were observed when compared to LPS-treated endothelial cells. These data demonstrated that the MS-MSC groups had potential therapeutic effects on the LPS-treated ECs; these results might be useful in the treatment of ARDS.

## 1. Introduction

To date, the emerging virus SARS-CoV-2 is causing a worldwide public health emergency; 17% critically ill patients developed acute respiratory distress syndrome (ARDS) [1]. Despite numerous efforts towards reducing mortality in established ARDS, in hospital mortality still remains near 40% [2]. The main pathophysiology associated with ARDS in critically ill patients is the failure of pulmonary microvascular endothelium barrier integrity [3]. Therefore, maintaining the integrity of the endothelium barrier is critical for ARDS treatment.

Mesenchymal stem cell (MSC) therapy is a potential method to treat ARDS [4], and our previous studies [5, 6] have shown concrete benefits both *in vitro* and *in vivo*. However, clinical trials of allogeneic MSC transplantation have provided conflicting evidences. In one trial, MSC treatment in patients with ARDS produced a short-term improvement in oxygenation [7]. Yet, another trial demonstrated no significant difference in the 28-day mortality between patients treated with MSCs and a control group (30% in the MSC group versus 15% in the placebo group) [8]. When injected intravenously, MSCs preferentially homed to the lungs and engrafted at sites of injury in the pulmonary microvascular endothelium layer [9]. The therapeutic function of MSCs presents from the beginning of their engraftment on the endothelium layer to their merger with the layer [10]. During this period, MSCs are not only affected by biochemical factors but also by different kinds of mechanical stimulation coming from the microenviroment they have lived in [11]. Better integration of experimental and clinical data could provide further insight into the use of MSC-based therapy in this setting.

Mechanical stimulation on the lung tissue exists constantly in physiological and pathological states, such as ARDS [12, 13]. When utilized with mechancial ventilation to maintain essential oxygenation of ARDS patients, mechanical stimulation conducted through the lung tissue microenvironment varies from mild to severe levels and generates different degrees of lung compliance [14, 15]. Mechanical stretch may approximate the mechanical ventilation with low tidal volumes that are commonly used in the lung-protective mechanical ventilation required to treat ARDS previously [16] and nowadays [17]. When MSCs are introduced into the lung microenvironment to treat ARDS, they have to encounter different degrees of mechanical stimulation. Evidences have shown that mechanical stimulation can affect behavior of MSCs, such as morphology [18], adhension [19], and differentiation [20, 21]. Therefore, mechanical stretch on MSCs could play an important role on the treatment of LPS-induced EC injuries.

The aim of the this study was to present evidences of MS-MSC therapeutic effects on EC injuries treated by LPS. To test this hypothesis, we conducted a cocultured model of the EC and MSC medium obtained from MSCs with or without mechanical stretch. And, we evaluated the repair ability of the medium from MSCs or MS-MSCs on LPS-induced EC injuries.

## 2. Materials and Methods

### 2.1. Mesenchymal Stem Cell Culture and Mechanical Stretch

First passage human bone marrow mesenchymal stem cells (MSCs) were obtained from ScienCell Research Laboratories (San Diego, California, USA). The cells were characterised by the supplier. MSCs were maintained in the mesenchymal stem cell medium (MSCM; 5%FBS, 1% mesenchymal stem cell growth supplement, and 1% penicillin/streptomycin solution). The media were purchased from ScienCell Research Laboratories (San Diego, California, USA). Cells were cultured at 37°C in an incubator with an atmosphere of 5% CO_2_ air. Every 3 to 5 days, cells were passaged when they reached 70-80% confluency and passages from 3 to 8 of the cells were used for all experiments.

MSCs were preconditioned by mechanical stretch (MS) *in vitro* with a BioFlex strain unit (BioFlex, Flexcell International Corporation, Hillsborough, NC, USA) as described previously [22]. MSCs were seeded onto a six-well plate containing flexible collagen type I-coated silicone rubber membranes at the bottom of each well and incubated at 37°C in 5% CO_2_ atmosphere with 95% humidity (BioFlex, Flexcell International Corporation, Hillsborough, NC, USA). MSCs were cultured for 3 or 5 days to reach 70-80% confluency and subjected to mechanical stretch of 10% or 20% elongation for 24 h or 48 h using a computer-controlled vacuum stretch apparatus (FX-5000 Tension Plus System, Flexcell International Corporation, Hillsborough, NC, USA). The untreated MSC group did not receive mechanical stretch and was incubated in the same incubator. MSCs and supernatant from all groups were collected at scheduled time points and prepared for use in this study. Supernatants from the stretched MSC and control groups were collected and centrifugated to remove dead cells and cell debris.

### 2.2. Endothelial Cell and Dermal Fibroblast Culture

First passage human pulmonary microvascular endothelial cells (ECs) and human dermal fibroblasts (HDF) were obtained from ScienCell Research Laboratories (San Diego, California, USA), and the cells were cultured in an endothelial cell medium (ECM; 5%FBS, 1% endothelial cell growth supplement, and 1% penicillin/streptomycin solution) and fibroblast medium (FM; 5%FBS, 1% fibroblast growth supplement, and 1% penicillin/streptomycin solution), respectively. All mediums were purchased from ScienCell Research Laboratories (San Diego, California, USA). Cells were cultured at 37°C in the incubator with an atmosphere of 5% CO_2_ air. Every 3 to 5 days, cells were passaged when they reached 70-80% confluency.

### 2.3. Endothelial Cell Intervention with LPS and Coculture System

ECs at a density of 50,000 per well were seeded in the upper chambers (0.4 *μ*m pore size polyester membrane from Corning, Inc.) and cultured for 2 to 3 days to produce a confluent monolayer, and MSCs were seeded in the lower chambers,. Then, cells were treated with LPS (100 ng/mL, Sigma) for 6 hours before permeability was tested, as previously described [23]. After adding 10 *μ*L 40 kDa fluorescein isothiocyanate- (FITC-) Dextran (Sigma-Aldrich) to each upper insert and incubating for 40 minutes in an incubator, 100 *μ*L medium from the upper and lower chambers was withdrawn. Then, the medium was transferred to a 96-well plate and read using excitation and emission wavelengths of 490 nm and 530 nm, respectively.

### 2.4. Morphology Assessment of Mesenchymal Stem Cells

To observe cell morphology, cells were stained with Wright-Giemsa stain (Sigma Aldrich) according to the manufacturer's protocols as previously described [24]. After air drying the wells, MSCs were inspected under a light microscope (Olympus, Tokyo, Japan).

### 2.5. Cell Proliferation Assay

Untreated and mechanically stretched MSCs were seeded at 2000 cells per well onto 96-well plates and cultured in an incubator with a humidified atmosphere of 5% CO_2_ at 37°C. 10 *μ*L of Cell Counting Kit-8 (CCK-8) solution (Beyotime, China) was added per well, and cells were cultured for 1 hour at 37°C, before measuring absorbance at 450 nm with a microplate reader.

### 2.6. Identification of MSCs by Flow Cytometry

Untreated and mechanically stretched MSCs were identified by flow cytometry (BD Bioscience, San Diego, CA) as described previously [25]. Harvested MSCs were washed with PBS and resuspended to 1 × 10^6^ cells/mL, and 100 *μ*L of cell suspension was incubated with fluorescein-conjugated monoclonal antibodies against CD90, CD29, and CD45 (BD Pharmingen, San Diego, CA), respectively. Samples were mixed in the dark for 20 minutes, then resuspended and centrifuged at 1000 rpm for 5 minutes at room temperature. Supernatants were removed, and cells were resuspended with PBS to 200 *μ*L for flow cytometry analysis.

### 2.7. Enzyme-Linked Immunosorbent Assay

The supernatants from all MSC groups were collected and centrifuged to remove cell fragments. Levels of tumor necrosis factor (TNF-*α*), interleukin-6 (IL-6), and interleukin-10 (IL-10) in the culture medium were detected by ELISA (ExCellBio, Shanghai, China). All tests were performed according to the manufacturer's instructions. All samples were examined in duplicate.

### 2.8. Endothelial Permeability Examination

ECs were seeded in the upper chamber in 24-well culture plates (0.4 *μ*m pore size polyester membrane from Corning, Inc.) and cultured for 2 to 3 days to produce a confluent monolayer. Then, cells were treated with LPS (100 ng/mL, Sigma) for 6 hours before permeability was tested, as previously described [23]. After adding 10 *μ*L 40 kDa fluorescein isothiocyanate- (FITC-) Dextran (Sigma-Aldrich) to each upper insert and incubating for 40 minutes in an incubator, 100 *μ*L medium from the upper and lower chambers was withdrawn. Then, the medium was transferred to a 96-well plate and read using excitation and emission wavelengths of 490 nm and 530 nm, respectively.

### 2.9. Apoptosis of Pulmonary Microvascular Endothelial Cells

An Annexin V-FITC Assay Kit (Sigma–Aldrich) was used to assess the percentage of ECs undergoing apoptosis, according to the manufacturer's instructions. ECs were harvested and washed with PBS and suspended in 1x binding buffer at a cell concentration of 1 × 10^6^ cells/mL. Then, 10 *μ*L propidiumiodide solution (PI) and 5 *μ*L annexin V-FITC conjugate (annexin V) were added to each sample and gently mixed. After 10 minutes incubation in the dark at room temperature, samples were analyzed using a flow cytometer (BD Biosciences, USA).

### 2.10. Western Blotting Analysis

Western blotting was used to detect the expression of VE-cadherin and Connexin-43 on ECs as previously described [26]. Total proteins from ECs after different treatments were extracted with RIPA lysis buffer (Beyotime Institute of Biotechnology, Shanghai, China) supplemented with 1 mmol/L phenylmethylsulfonyl fluoride (PMSF), and then separated with 10% sodium dodecyl sulphate-polyacrylamide gel electrophoresis and transfered onto polyvinylidene fluoride membranes (Beyotime, China). Afterwards, membranes were blocked in 3% BSA for 2 hours at room temperature and incubated at 4°C overnight with primary antibodies against VE-cadherin (Abcam) or Connexin-43 (Cell Signaling Technology). The next day, membranes were washed in TBS-T and incubated in HRP-conjugated secondary antibody (Boster biotechnology, Wuhan, China) for 1 hour at room temperature. Then, ECL (Beyotime, China) was applied to detect the bands with a chemiluminescence imaging system (ChemiQ 4800mini; Ouxiang, China).

### 2.11. Immunofluorescence Staining

In a transwell system, ECs were seeded on the upper inserts and cultured to form a confluent monalayer for 3 or 5 days. After treating with LPS for 6 hours, cells were then washed with cold PBS and fixed with 4% paraformaldehyde for 10 minutes. Samples were permeabilized with 0.25% Triton X-100 for 10 minutes, blocked with 1% bovine serum albumin (BSA), and incubated overnight with VE-cadherin primary antibody (AB) (1 : 200 rabbit polyclonal anti-VE-cadherin) (Abcam, ab18058, Ireland) at 4°C. After incubation for 6 hours, samples were incubated with a secondary FITC-conjugated goat anti-rabbit IgG (1 : 700 Alexa Fluor 488 IgG) (Biosciences, Ireland) and stained with (VWR, Ireland) for 1 h at room temperature. Cell nucleis were stained with DAPI (VWR, Ireland) for 1 min at room temperature, washed in PBS, and imaged using confocal microscopy (Leica SP8, Ireland).

### 2.12. Statistical Analyses

Statistical analyses were performed using the SPSS 20.0 software package (SPSS Inc., Chicago, IL, USA). Results were presented as the mean ± standard deviation. Group comparison was analyzed by one-way analysis of variance, followed by Tukey's test. *p* < 0.05 was considered statistically significant.

## 3. Results

### 3.1. MSCs Improved Paracellular Permeability of LPS-Induced EC Injury

**Figure 1:**
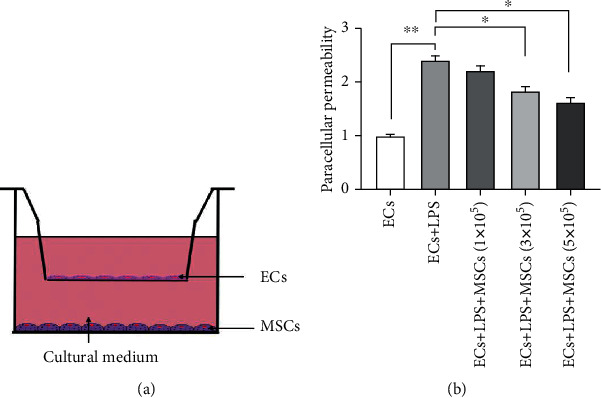
Paracellular permeability of ECs induced by LPS. (a) Schematic view of the transwell coculture system. (b) Effect of adding different numbers of MSCs on the paracellular permeability of ECs. Statistically significant differences were presented (*n* = 3; ^∗^*p* < 0.05, ^∗∗^*p* < 0.01). ARDS: acute respiratory distress syndrome; MSCs: mesenchymal stem cells; ECs: endothelial cells; LPS: lipopolysaccharide.

To determine if MSCs protected ECs from LPS-induced injury, we used a transwell coculture system (Figure 1(a)) to assess paracellular permeability when MSCs were added at varying seeding concentrations, from 1 × 10^5^ cells per well to 5 × 10^5^ cell per well. Permeability significantly decreased when MSCs were plated at 3 × 10^5^ cell per well (Figure 1(b); ^∗^*p* < 0.05) and decreased further as the density of MSCs increased. This suggested that the therapeutic effect of MSCs on endothelial cell permeability improved as the density of MSCs increased.

### 3.2. Description of the Mechanical Stretch Method of MSCs

**Figure 2:**
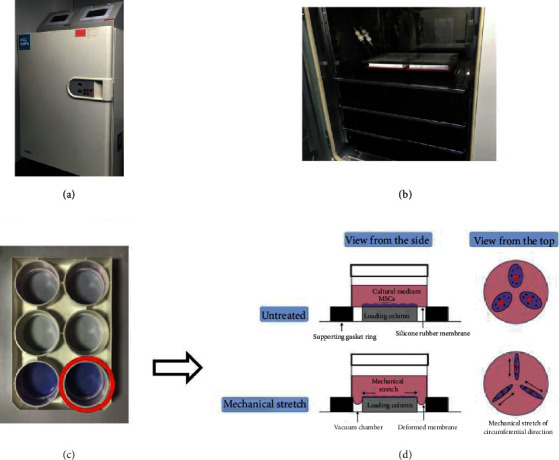
Illustration of method used to precondition MSCs by mechanical stretching. MSCs were seeded onto a six-well plate with collagen type I-coated flexible silicone rubber membranes at the bottom of each well in a mechanical stretch system. (a) Outside view of the mechanical stretch system. (b) Inside view of the mechanical stretch system. (c) Example of a six-well mechanical stretch plate. (d) Illustration of a well under mechanical stretch.

MSCs were seeded on six-well mechanical stretch plates with collagen type I-coated flexible silicon rubber membranes placed at the bottom of each well and were preconditioned by mechanically stretching these plates during cell culture (Figures 2(a) and 2(b)). An example of a six-well mechanical stretch plate is presented in Figure 2(c). MSCs were plated on the silicon rubber membrane and stained with Wright-Giemsa stain. A schematic view of a well under mechanical stretch is presented in Figure 2(d). The first column shows the side view of two wells containing either untreated or mechanically stretched MSCs. The second column presents an illustration of untreated or mechanically stretched MSCs, respectively.

### 3.3. Mechanical Stretch Affected Morphology and Proliferation of MSCs

**Figure 3:**
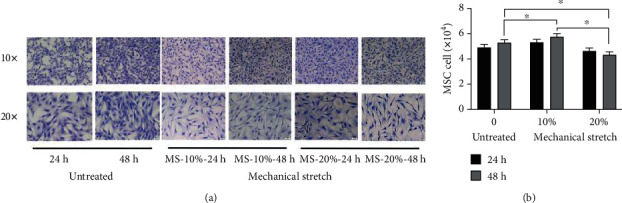
Effects of MS on MSC morphology and proliferation. (a) Changes to MSCs morphology with or without MS were presented in a time and magnitude dependent manner. (b) MSC cell number after MS. Statistically significant differences are presented (*n* = 3; ^∗^*p* < 0.05). MS: mechanical stretch.

MSCs were seeded on the six-well plates and subjected to different categories of mechanical stretch (MS), including untreated, 10% MS for 24 hours (MS-10%-24 h), 10% MS for 48 hours (MS-10%-48 h), 20% MS for 24 hours (MS-24%-24 h), and 20% MS for 48 hours (MS-20%-48 h). Morphological differences were observed following treatment (Figure 3(a)). Cells in all groups remained firmly adhered to the seeding surface. Compared to untreated MSCs, the MS-MSCs showed signs of atrophy, appearing thinner and flattened, and have increasingly shrunk in a time- and magnitude-dependent manner. Moreover, cell proliferation significantly increased in the MS-10% groups (Figure 3(b); ^∗^*p* < 0.05) but decreased in the MS-20% groups (^∗∗^*p* < 0.01). Proliferation in the MS-20%-48 h group was significantly lesser than that in the MS-10%-48 h group (^∗^*p* < 0.05). These data suggest that MS affected the morphology and proliferation of MSCs significantly.

### 3.4. Mechanical Stretch Did Not Alter Expression of Surface Markers on MSCs

**Figure 4:**
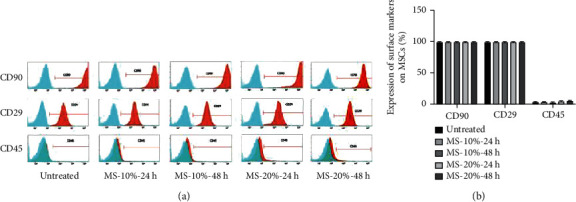
Identification of mesenchymal stem cells (MSCs). Immunophenotypic analysis of surface markers by flow cytometry. (a) Cyan peak: isotype control; red peak: sample. (b) Effects of mechanical stretching on the expression of MSC surface markers CD90, CD29, and CD45 as analyzed by flow cytometry.

Surface markers on MSCs served as an index parameters for the identification of MSCs [25]. To determine if surface marker expression changed when MSCs were preconditioned to mechanical stretch, we used flow cytometry to analyze major surface markers of MSCs for identification, such as CD90, CD29, and CD45 (Figure 4). The results showed no statistical change in the expression with high levels of CD90 and CD29 expressions on nearly 99% of cells in all treatment groups and low levels of CD45 expression on fewer than 5% of cells for all treatment groups. The results suggest that MS did not alter the expression of surface markers.

### 3.5. Mechanical Stretch Affected the Production of Inflammation Mediators in MSCs

**Figure 5:**
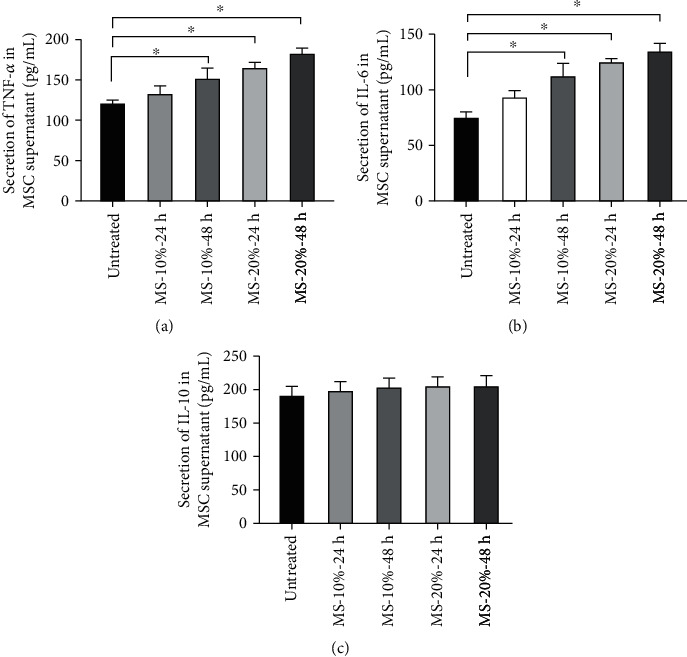
Effects of MS on inflammation mediators secreted by MSCs. ELISA was used to detect the concentrations of (a) TNF-*α*, (b) IL-6, and (c) IL-10 in the supernatant of the MSC groups (*n* = 3; ^∗^*p* < 0.05). ELISA: enzyme-linked immunosorbent assay; TNF-*α*: tumor necrosis factor-*α*; IL-6: interleukin-6; IL-10: interleukin-10.

Studies have shown that MS can induce biological function change [27]. To evaluate the effects of MS on the inflammatory function of MSCs, we examined the inflammatory mediators TNF-*α*, IL-6, and IL-10 presented in the MSC supernatants by enzyme-linked immunosorbent assay. The results showed that TNF-*α* and IL-6 increased statistically as time and magnitude of mechanical stretch increased (Figures 5(a) and 5(b); ^∗^*p* < 0.05), but the MS-10%-24 h group did not produce significant differences when compared to the untreated MSC group. However, IL-10 did not significantly change in all groups (Figure 5(c)). These results showed that MS could statistically increase the TNF-*α* and IL-6 levels.

### 3.6. MS-MSCs Decreased Paracellular Permeability of Pulmonary Microvascular Endothelium Barrier Injured by LPS

**Figure 6:**
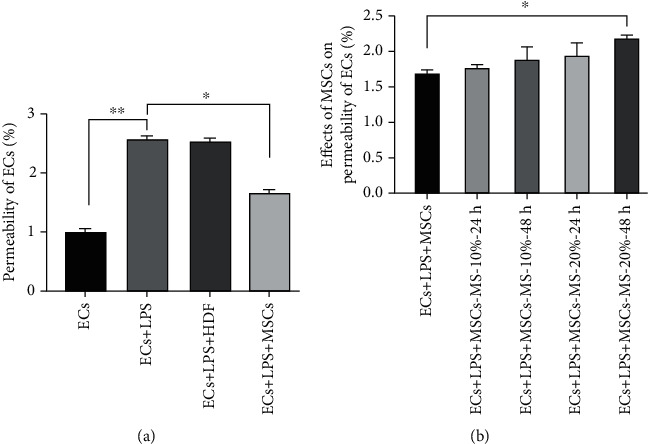
Effects of MS-MSCs on permeability of ECs treated with LPS. Permeability of ECs induced by LPS was detected using FITC-Dextran (*n* = 3; ^∗^*p* < 0.05, ^∗∗^*p* < 0.01). HDF: human dermal fibroblasts; FITC: fluorescein isothiocyanate; ECs: endothelial cells; MS: mechanical stretch.

Evaluation of paracellular permeability is a critical step in assessing the integrity of the pulmonary microvascular endothelium barrier [28]. We introduced a transwell coculture system to evaluate the effects of MS-MSCs on the paracellular permeability of LPS-treated ECs. Treatment with LPS significantly increased the paracellular permeability of the pulmonary microvascular endothelium barrier (Figure 6(a); ^∗∗^*p* < 0.01). And MSCs significantly attenuated the increased paracellular permeability induced by LPS (Figure 6(a); ^∗^*p* < 0.05), while HDF showed no effect on the increased permeability. These results suggested that MS-MSCs attenuated the increased permeability of LPS-treated ECs.

### 3.7. MS-MSCs Attenuated Apoptosis of ECs Induced by LPS

**Figure 7:**
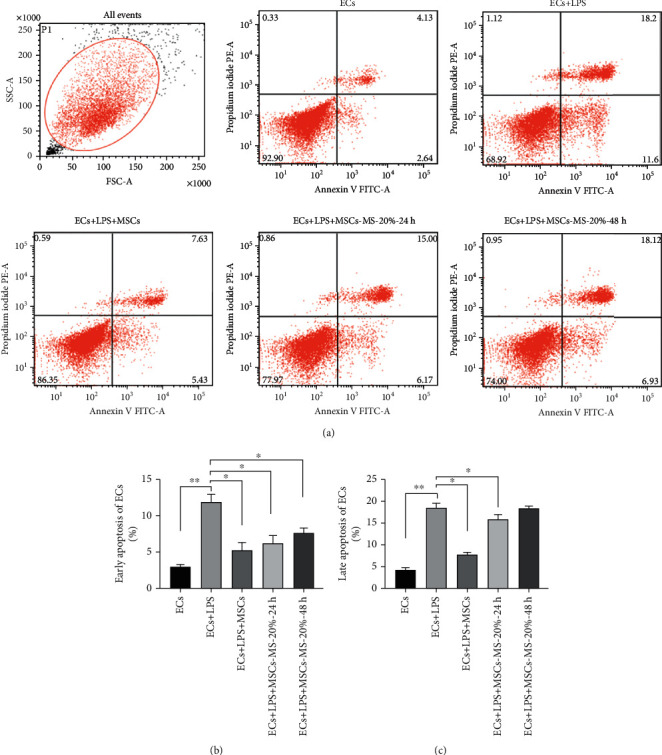
Effects of MS-MSCs on apoptosis of ECs. (a) Flow cytometric analysis of ECs was performed to assess apoptotic and necrotic cells. (b) Early apoptotic ECs stained only with annexin V were presented in the lower right quadrant. (c) Late apoptotic or necrotic ECs stained both with annexin V and PI were presented in the upper right quadrant (*n* = 3; ^∗^*p* < 0.05, ^∗∗^*p* < 0.01). ECs: endothelial cells; PI: propidium iodide.

LPS is a useful agent to induce injury and apoptosis on pulmonary microvascular endothelial cells [29]. In this study, we applied the flow cytometry to evaluate the effect of MSCs on apoptosis of ECs treated with LPS (Figure 7(a)). LPS could significantly induce the apoptosis of ECs both in early and late states (Figures 7(b) and 7(c); ^∗∗^*p* < 0.01), but MSCs decreased the apoptosis of LPS-treated ECs (^∗^*p* < 0.05). Furthermore, the MS-20%-24 h MSC group could significantly attenuate both early and late apoptosis of ECs (^∗^*p* < 0.05), similar to the untreated MSC group (Figure 7(b)). However, the MS-20%-48 h group significantly decreased early apoptosis (^∗^*p* < 0.05) but not late apoptosis of ECs, although it showed a trend towards attenuating apoptosis (Figure 7(c)).

### 3.8. MS-MSCs Restored Intercellular Junction Proteins

**Figure 8:**
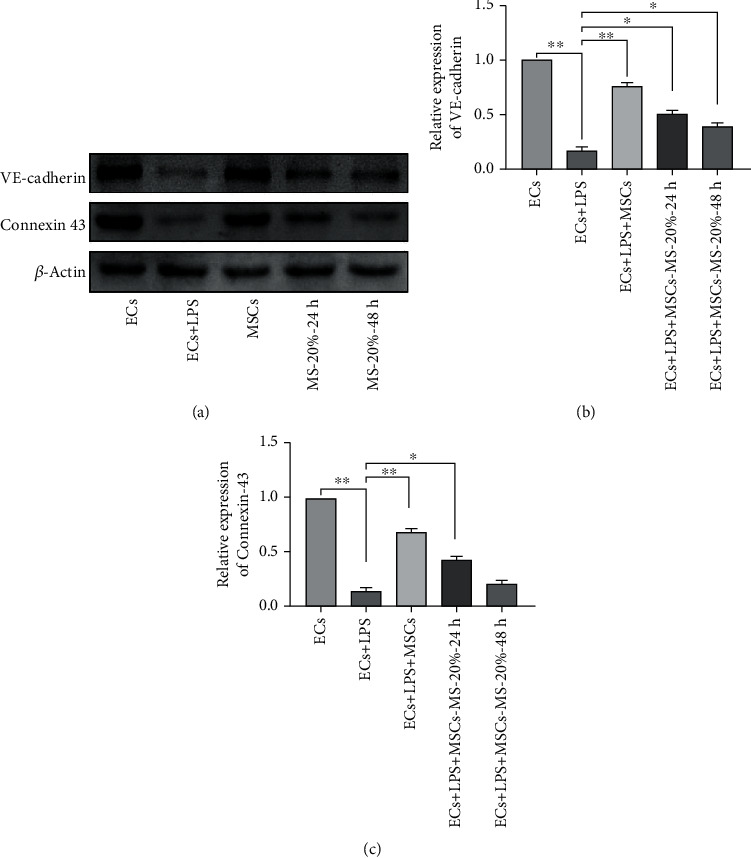
Effects of MS-MSCs on endothelial intercellular junction protein. Western blotting presented protein expression of VE-cadherin and Connexin-43 (a). Compared with the LPS-treated EC group, the MSC and MS-MSC groups increased VE-cadherin (b) and Connexin-43 (c) expressions (*p* < 0.01). VE-cadherin: vascular endothelial cadherin.

**Figure 9:**
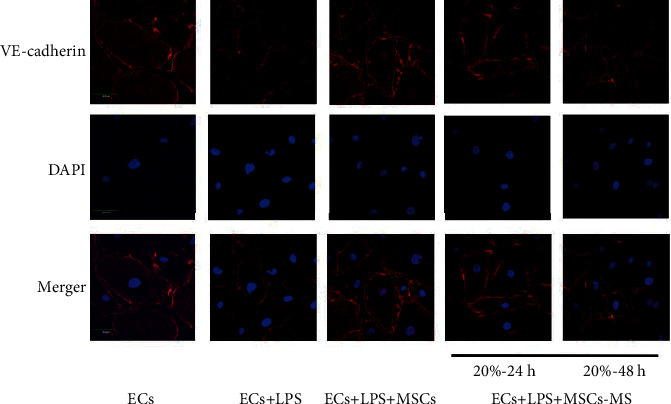
Effects of MS-MSCs on VE-cadherin. VE-cadherin was detected by immunofluorescent staining and observed by confocal microscopy. VE-cadherin: vascular endothelial cadherin; DAPI: 4′,6-diamidino-2-phenylindole.

Intercellular junction proteins play an important role in maintaining the integrity of the pulmonary microvascular endothelium barrier. VE-cadherin [3] and Connexin-43 [30] present critical effects on regulating the permeability of the barrier. To investigate the effects of MS-MSCs on endothelium barrier integrity, we examined the expression of these two key proteins. Compared with the LPS-treated ECs, MSCs increased the expression of VE-cadherin and Connexin-43 (Figures 8(b) and 8(c); ^∗∗^*p* < 0.01). We also applied immunofluorescent staining to detect the protein expression of ECs and observed the cells under confocal microscopy. The results showed that VE-cadherin located on the surface of ECs were destroyed after LPS treatment, thus leading to the loss of integrity of the pulmonary microvascular endothelium barrier (Figure 9). These data indicated that MS-MSCs restored the intercellular junction of LPS-treated ECs.

## 4. Discussion

ARDS is the leading cause of mortality in ICU patients [31] and featured with acute diffuse lung injury, which results in severely injured lung compliance and increased pulmonary vascular permeability [3]. MSC is a promising method to restore endothelial function [32], but when engrafted on the alveolocapillary barrier, the efficacy of MSCs under mechanical stretch in the context of decreased lung compliance remains unproven. Our study tried to reveal the effect of mechanically stretched MSCs on restoring the injured alveolocapillary barrier. We applied a mechanical stretch system to simplify yet still mimic the mechanical microenviroments present within the lung in a simplified way. We demonstrated that mechanical stretch could impact MSC morphology and biological function in a time- and magnitude-dependent manner and that MS-MSCs could restored the increased permeability of endothelial cells induced by LPS.

The alveolocapillary barrier provides an essential function in regulating the diffusive exchange of molecules. Loss of barrier integrity could lead to excessive leakage of fluid and proteins from the vasculature to the alveoli, producing the pulmonary edema common in ARDS [33]. Sepsis plays a major role in extrapulmonary edema, and the endothelial barrier stands as the first line of defense in keeping LPS out of the vascular system [34]. Studies have demonstrated that endothelial injury is a more important consideration in extrapulmonary ARDS than pulmonary ARDS [35, 36]. As a major factor driving sepsis and lethal septic shock, LPS has been studied in in vivo, in vitro, and ex vivo settings [37, 38]. Hereby, we adopted LPS and a transwell coculture system to investigate the effects of MSCs on the permeability of the alveolocapillary barrier. We found that increased permeability by LPS was significantly decreased by MSCs as the cell density increased accordingly.

Manipulation of mesenchymal stem cell functions is important for tissue engineering and regenerative medicine. Heterogeneous mechanical properties of the alveolocapillary barrier in ARDS caused a complicated microenviroment for the engraftment of MSCs [36]. So, we used an apparatus to mimic and simplify the mechanical properties within the lung tissue in clinical field, as 10% mechanical stretch for physical stimulation and 20% for severe pathological status. Our previous research had applied this method and acquired positive therapeutic results of pulmonary fibrosis investigation [22]. In this study, we tried to discover evidences of mechanically stretched MSCs in restoring increased permeability of endothelial barrier induced by LPS. While MSCs injected via the bloodstream preferred to engraft on and merge into the injured sites of the pulmonary microvascular endothelial barrier [9]. Studies have proved that MSCs can coexist with endothelial cells and other kind of cells in the barrier for about 24 to 48 hours. [35, 39] Therefore, we investigated MSCs under these time durations of 10% and 20% mechanical stretch as used previously [22]. We demonstrated that mechanical stretch affects cell morphology and cell proliferation, suggesting that mechanical stretch is important for the maintenance of MSC functions.

The most attractive charateristics of MSCs are the stemness and self-renewal. These properties make them a promising therapeutic tool in many clinical field, such as the kidney [40], liver [41], and lung [42]. The stemness of MS-MSCs is analyzed by the expression of surface markers. When under the mechanical stretch modes in this study, whether MSCs could maintain their stemness is crucial for the LPS-treated EC therapy. The expression of CD90, CD29, and CD45 did not change with the intervention of different mechanical stretch patterns. All groups exhibit similar expression of the surface markers. The results indicate that, in 48 h with the maximum MS-20%, the MSCs could maintain the stemness.

Increased permeability resulted from the disruption of the pulmonary endothelial barrier [36]. EC apoptosis played a vital role in EC barrier integrity [43] We have been proved that MSCs without mechanical stretch could repair the injured EC barrier in a cell density-dependent manner. Nowadays, tissue engineering shows great impact on MSC therapy and achieved great advances [44]. Mechanical stretch, a method to mimic the mechanical properties of the lung tissue, played an important role in the microenviroment where MSCs engrafted. Novel strategies to isolate the mechanical factor could shed some light on MSC application. Our results indicated that LPS can induce both early and late apoptosis, and mechanically stretched MSCs decreased EC apoptosis but decreased slightly as the time and magnitude of MS increased. Thus, mechanical stretch may account for the restoring effect on the injured EC barrier through EC apoptosis.

Constant remodeling of intercellular junctions to regulate the transendothelial permeability is essential in maintaining endothelium barrier functions. Treatment with LPS can also alter the apoptotic status of endothelial barrier cells and badly damage the paracellular architecture of causing the endothelial barrier to function abnormally and producing pulmonary edema [43]. Of the intercellular juntion proteins, VE-cadherin and Connexin-43 are vital for the barrier integrity and could act as index parameters to evaluate the disruption of barrier [45]. The results presented that endothlial barrier integrity was severely damaged by LPS, but MS-MSCs increased VE-cadherin and Connexin-43 expressions, which favored the integrity of the endothelial barrier. Therefore, MS-MSCs restored the increased permeability of the endothelial cell partially by remodeling of VE-cadherin and Connexin-43.

The following limitations to this research should be noted. The mechanical stretch system that we used in this study applies a vacuum to generate mechanical stretch, but as the culture medium is flowing between the MSCs, other categories of mechanical stimulations, including shear force and pressure force, are not absent and may influence cells in some level. These forces could also produce a biological response and may thus affect MSC function. However, by their nature, these three forces are often mixed together and are difficult to study separately. This could be addressed in future studies.

## 5. Conclusions

In conclusion, our experiments reveal that MS-MSCs statistically improved the increased permeability of the EC barrier induced by LPS, through decreasing the apoptosis of ECs and increasing the remodeling of intercellular junctions. These findings provide additional *in vitro* evidences for the therapeutic potential of MSCs and may be useful for the clinical utilization of MSCs.

## Data Availability

The data used to support the findings of this study are included within the article.

## Abbreviations

ECs: Endothelial cells
ARDS: Acute respiratory distress syndrome
MSCs: Mesenchymal stem cells
MS: Mechanical stretch
LPS: Lipopolysaccharide
ELISA: Enzyme-linked immunosorbent assay
FITC: Fluorescein isothiocyanate
TNF-*α*: Tumor necrosis factor-alpha
IL: Interleukin
VE-cadherin: Vascular endothelial-cadherin.

## References

[1] Chen N., Zhou M., Dong X. (2020). Epidemiological and clinical characteristics of 99 cases of 2019 novel coronavirus pneumonia in Wuhan, China: a descriptive study. *The Lancet*.

[2] Cummings M. J., Baldwin M. R., Abrams D. (2020). Epidemiology, clinical course, and outcomes of critically ill adults with COVID-19 in New York City: a prospective cohort study. *The Lancet*.

[3] Radeva M. Y., Waschke J. (2018). Mind the gap: mechanisms regulating the endothelial barrier. *Acta Physiologica*.

[4] Laffey J. G., Matthay M. A. (2017). Fifty years of research in ARDS. Cell-based therapy for acute respiratory distress syndrome. Biology and potential therapeutic value. *American Journal of Respiratory and Critical Care Medicine*.

[5] Hu S., Li J., Xu X. (2016). The hepatocyte growth factor-expressing character is required for mesenchymal stem cells to protect the lung injured by lipopolysaccharide in vivo. *Stem Cell Research & Therapy*.

[6] Meng S.-S., Guo F. M., Zhang X. W. (2018). MTOR/STAT-3 pathway mediates mesenchymal stem cell-secreted hepatocyte growth factor protective effects against lipopolysaccharide-induced vascular endothelial barrier dysfunction and apoptosis. *Journal of Cellular Biochemistry*.

[7] Zheng G., Huang L., Tong H. (2014). Treatment of acute respiratory distress syndrome with allogeneic adipose-derived mesenchymal stem cells: a randomized, placebo-controlled pilot study. *Respiratory Research*.

[8] Matthay M. A., Calfee C. S., Zhuo H. (2019). Treatment with allogeneic mesenchymal stromal cells for moderate to severe acute respiratory distress syndrome (START study): a randomised phase 2a safety trial. *The Lancet Respiratory Medicine*.

[9] Sohni A., Verfaillie C. M. (2013). Mesenchymal stem cells migration homing and tracking. *Stem Cells International*.

[10] Steingen C., Brenig F., Baumgartner L., Schmidt J., Schmidt A., Bloch W. (2008). Characterization of key mechanisms in transmigration and invasion of mesenchymal stem cells. *Journal of Molecular and Cellular Cardiology*.

[11] Grune J., Tabuchi A., Kuebler W. M. (2019). Alveolar dynamics during mechanical ventilation in the healthy and injured lung. *Intensive Care Medicine Experimental*.

[12] Knudsen L., Ochs M. (2018). The micromechanics of lung alveoli: structure and function of surfactant and tissue components. *Histochemistry and Cell Biology*.

[13] Goetzke R., Sechi A., De Laporte L., Neuss S., Wagner W. (2018). Why the impact of mechanical stimuli on stem cells remains a challenge. *Cellular and Molecular Life Sciences*.

[14] Mora Carpio A. L., Mora J. I. (2020). *Ventilator Management*.

[15] The Acute Respiratory Distress Syndrome Network (2000). Ventilation with lower tidal volumes as compared with traditional tidal volumes for acute lung injury and the acute respiratory distress syndrome. *The New England Journal of Medicine*.

[16] Spinelli E., Mauri T., Beitler J. R., Pesenti A., Brodie D. (2020). Respiratory drive in the acute respiratory distress syndrome: pathophysiology, monitoring, and therapeutic interventions. *Intensive Care Medicine*.

[17] Alhazzani W., Møller M. H., Arabi Y. M. (2020). Surviving sepsis campaign: guidelines on the management of critically ill adults with Coronavirus Disease 2019 (COVID-19). *Intensive Care Medicine*.

[18] Maul T. M., Chew D. W., Nieponice A., Vorp D. A. (2011). Mechanical stimuli differentially control stem cell behavior: morphology, proliferation, and differentiation. *Biomechanics and Modeling in Mechanobiology*.

[19] Li C. W., Lau Y. T., Lam K. L., Chan B. P. (2020). Mechanically induced formation and maturation of 3D-matrix adhesions (3DMAs) in human mesenchymal stem cells. *Biomaterials*.

[20] O'Cearbhaill E. D., Punchard M. A., Murphy M., Barry F. P., McHugh P. E., Barron V. (2008). Response of mesenchymal stem cells to the biomechanical environment of the endothelium on a flexible tubular silicone substrate. *Biomaterials*.

[21] Nieponice A., Maul T. M., Cumer J. M., Soletti L., Vorp D. A. (2007). Mechanical stimulation induces morphological and phenotypic changes in bone marrow-derived progenitor cells within a three-dimensional fibrin matrix. *Journal of Biomedical Materials Research Part A*.

[22] Zhang R., Pan Y., Fanelli V. (2015). Mechanical stress and the induction of lung fibrosis via the midkine signaling pathway. *American Journal of Respiratory and Critical Care Medicine*.

[23] Armstrong S. M., Khajoee V., Wang C. (2012). Co-regulation of transcellular and paracellular leak across microvascular endothelium by dynamin and Rac. *The American Journal of Pathology*.

[24] Liu F., Lee J. Y., Wei H. (2010). FIP200 is required for the cell-autonomous maintenance of fetal hematopoietic stem cells. *Blood*.

[25] Dominici M., le Blanc K., Mueller I. (2006). Minimal criteria for defining multipotent mesenchymal stromal cells. The International Society for Cellular Therapy position statement. *Cytotherapy*.

[26] Chen Q. H., Liu A. R., Qiu H. B., Yang Y. (2015). Interaction between mesenchymal stem cells and endothelial cells restores endothelial permeability via paracrine hepatocyte growth factor in vitro. *Stem Cell Research & Therapy*.

[27] Saparov A., Ogay V., Nurgozhin T., Jumabay M., Chen W. C. W. (2016). Preconditioning of human mesenchymal stem cells to enhance their regulation of the immune response. *Stem Cells International*.

[28] Pati S., Gerber M. H., Menge T. D. (2011). Bone marrow derived mesenchymal stem cells inhibit inflammation and preserve vascular endothelial integrity in the lungs after hemorrhagic shock. *PLoS One*.

[29] Meng F., Meliton A., Moldobaeva N. (2015). Asef mediates HGF protective effects against LPS-induced lung injury and endothelial barrier dysfunction. *American Journal of Physiology-Lung Cellular and Molecular Physiology*.

[30] Parthasarathi K. (2012). Endothelial connexin43 mediates acid-induced increases in pulmonary microvascular permeability. *American Journal of Physiology-Lung Cellular and Molecular Physiology*.

[31] Bellani G., Laffey J. G., Pham T. (2016). Epidemiology, patterns of care, and mortality for patients with acute respiratory distress syndrome in intensive care units in 50 countries. *JAMA*.

[32] Walter J., Ware L. B., Matthay M. A. (2014). Mesenchymal stem cells: mechanisms of potential therapeutic benefit in ARDS and sepsis. *The Lancet Respiratory Medicine*.

[33] Zayed Y., Askari R. (2019). *Respiratory Distress Syndrome*.

[34] Brooks D., Barr L. C., Wiscombe S., McAuley D. F., Simpson A. J., Rostron A. J. (2020). Human lipopolysaccharide models provide mechanistic and therapeutic insights into systemic and pulmonary inflammation. *European Respiratory Journal*.

[35] Matthay M. A., Anversa P., Bhattacharya J. (2013). Cell therapy for lung diseases. Report from an NIH-NHLBI workshop, November 13-14, 2012. *American Journal of Respiratory and Critical Care Medicine*.

[36] Jozwiak M., Teboul J. L., Monnet X. (2015). Extravascular lung water in critical care: recent advances and clinical applications. *Annals of Intensive Care*.

[37] Morrison D. C., Ryan J. L. (1987). mechanisms. *Annual Review of Medicine*.

[38] Yang Z., Sun D., Yan Z. (2014). Differential role for p120-catenin in regulation of TLR4 signaling in macrophages. *Journal of Immunology*.

[39] Ryan A. L., Ikonomou L., Atarod S. (2019). Stem cells, cell therapies, and bioengineering in lung biology and diseases 2017. An official American Thoracic Society workshop report. *American Journal of Respiratory Cell and Molecular Biology*.

[40] Peired A. J., Sisti A., Romagnani P. (2016). Mesenchymal stem cell-based therapy for kidney disease: a review of clinical evidence. *Stem Cells International*.

[41] Hu C., Zhao L., Duan J., Li L. (2019). Strategies to improve the efficiency of mesenchymal stem cell transplantation for reversal of liver fibrosis. *Journal of Cellular and Molecular Medicine*.

[42] Matthay M. A. (2008). Treatment of acute lung injury: clinical and experimental studies. *Proceedings of the American Thoracic Society*.

[43] Fan E., Brodie D., Slutsky A. S. (2018). Acute respiratory distress syndrome: advances in diagnosis and treatment. *JAMA*.

[44] Lane S. W., Williams D. A., Watt F. M. (2014). Modulating the stem cell niche for tissue regeneration. *Nature Biotechnology*.

[45] Mehta D., Malik A. B. (2006). Signaling mechanisms regulating endothelial permeability. *Physiological Reviews*.

